# Ranking distribution reveals opposite shifts in evenness and survival thresholds of phytoplankton under environmental stress

**DOI:** 10.1093/ismejo/wrag049

**Published:** 2026-03-10

**Authors:** Sisi Ye, Li Gao, Chao Chang, Xinyi Zhang, Yun Zhou, Man Xiao, Fang Yang, Ming Li

**Affiliations:** State Key Laboratory of Soil and Water Conservation and Desertification Control, College of Natural Resources and Environment, Northwest A&F University, Yangling, Shaanxi 712100, China; College of Soil and Water Conservation Science and Engineering (Institute of Soil and Water Conservation), Northwest A&F University, Yangling, Shaanxi 712100, China; Institute for Sustainable Industries and Liveable Cities, Victoria University, PO Box 14428, Melbourne, Victoria 8001, Australia; State Key Laboratory of Soil and Water Conservation and Desertification Control, College of Natural Resources and Environment, Northwest A&F University, Yangling, Shaanxi 712100, China; State Key Laboratory of Soil and Water Conservation and Desertification Control, College of Natural Resources and Environment, Northwest A&F University, Yangling, Shaanxi 712100, China; State Key Laboratory of Soil and Water Conservation and Desertification Control, College of Natural Resources and Environment, Northwest A&F University, Yangling, Shaanxi 712100, China; State Key Laboratory of Lake Science and Environment, Nanjing Institute of Geography and Limnology Chinese Academy of Sciences, Nanjing 210008, China; State Key Laboratory of Soil and Water Conservation and Desertification Control, College of Natural Resources and Environment, Northwest A&F University, Yangling, Shaanxi 712100, China; State Key Laboratory of Soil and Water Conservation and Desertification Control, College of Natural Resources and Environment, Northwest A&F University, Yangling, Shaanxi 712100, China

**Keywords:** phytoplankton, diversity index, temperature, nutrient, co-occurrence network, biomass

## Abstract

Evaluating phytoplankton diversity in response to environmental stress is important in the context of climate change and increasing anthropogenic activities. Here, we investigated the response of the phytoplankton diversity indices to temperature and nutrient gradients. Combining microcosm experiments and field investigations, we found that the minimum percentage threshold required for a taxon to survive (*a*) first increased and then decreased with increasing temperature, but decreased with increasing nutrient levels. In contrast, the evenness of the taxon distribution (*k*) showed an opposite trend to *a*. We identified that the index *a* showed a significant negative correlation with positive cohesion, the absolute value of negative cohesion (|negative cohesion|), and total cohesion under non-stress conditions for algal growth, whereas *k* showed the opposite pattern. However, the relationships between *a*, *k*, and the cohesion values were not significant under stress conditions. In addition, closeness centrality was positively correlated with inhibition rate, whereas weighted degree, eigenvector centrality, positive connectivity, and |negative connectivity| were negatively correlated with it under eutrophication at moderate temperatures. Moreover, a change in biomass was the dominant initial response of phytoplankton to short-term environmental stress. Our results also indicated that the proliferation ability of the algal community was inversely related to its evenness (*k* and Pielou index), and vice versa. These findings clarify how phytoplankton diversity indices respond to environmental stress and how this response is associated with community structure under climate change and anthropogenic activities. This understanding is critical for assessing the health of global aquatic ecosystems.

## Introduction

Phytoplankton are important primary producers in aquatic ecosystems [1, 2], and their community composition and diversity play a crucial role in stabilizing aquatic ecosystems [3, 4]. However, anthropogenically induced eutrophication and climate change threaten the stability of aquatic ecosystems by increasing phytoplankton biomass but reducing their diversity [5–7]. Therefore, revealing the effects and mechanisms of climate change and eutrophication on phytoplankton diversity is a core research topic in global change ecology. This knowledge will help assess the impacts of climate change and eutrophication on global aquatic ecosystems and identify countermeasures.

The response of phytoplankton to temperature and nutrient gradients can be revealed through indoor culture experiments or field investigations, providing insights into the effects of climate change and eutrophication on phytoplankton diversity. Culture experiments typically involve pre-culturing water samples from the field to obtain near-natural phytoplankton communities [8, 9]. In contrast, field surveys provide information on diverse phytoplankton communities [10–12]. Both culture experiments and field investigations have demonstrated how different phytoplankton communities respond to temperature and nutrient gradients [13, 14]. However, drawing a general conclusion is challenging due to variations in the composition of phytoplankton communities in various studies.

To address the above challenges, researchers have attempted to evaluate phytoplankton communities using diversity indices. However, the traditional phytoplankton diversity indices primarily relies on species richness or evenness [15, 16] and do not establish a one-to-one correspondence with phytoplankton community matrices. Thus, the traditional phytoplankton diversity index-based evaluation method is inadequate for detecting subtle changes in phytoplankton community composition. A recent study found that an exponential distribution function can effectively fit the relative abundance and biomass of phytoplankton. Based on this finding, they derived three indices, including *a*, *k*, and *N* to characterize community composition [17]. These three indices were considered to be the minimum percentage threshold required for a taxon, such as genus, to survive within the community, the evenness of the taxon distribution and the theoretical maximum number of taxa, respectively. Particularly, when the relative biomass or abundance of a taxon falls below the *a* value, this taxon is likely to become extinct. A higher *k* value indicates a greater difference in relative biomass or abundance between rare and dominant taxa, resulting in more uneven taxon distribution within a community. In natural ecosystems, the observed taxon richness may not exceed *N*. Although the response of these indices to temperature and nutrient gradients can largely reveal how phytoplankton communities react to climate and nutrient variations, their exact implications remain unclear.

The response of phytoplankton communities to temperature and nutrient gradients depends on the specific ecological niche of each phytoplankton species and their interspecific competition. Competition between two algal species can usually be simulated by the Lotka-Volterra competition model [18, 19] and the resource competition model [20]. The Lotka-Volterra model could also be extended to simulate competition among multiple species, but the extended model remains inadequate because it oversimplifies the complex interactions in natural algal communities [21, 22]. Co-occurrence network analysis has been widely used in recent years to analyze coexistence among multiple species [23, 24]. The topological characteristics of co-occurrence networks provide information about complexity and stability of communities [24, 25]. Thus, the topological characteristics of co-occurrence networks provide a strong matrix of indices for ecosystem function and integrity, which can be used as an important tool for studying the interrelationships among species in communities. If relationships between the topological characteristics of co-occurrence networks and diversity indices could be established, it would reveal how diversity responds to environmental change in terms of inter-species relationships. The variations in inter-species relationships could be inferred from changes in diversity indices, especially based on the ranking distribution of phytoplankton.

The relationship between phytoplankton diversity and productivity is another important issue in diversity research. Richness is one of the most direct and important indicators of species diversity [26]. The biomass of a phytoplankton community is limited when the species richness in the community is minimal, and it increases gradually as the richness of the phytoplankton community increases [14, 27]. However, higher species richness in a community does not always lead to higher biomass. A field investigation found that the shape of the productivity–diversity relationship (PDR) for marine phytoplankton was unimodal, meaning that with the gradual increase in phytoplankton abundance, productivity initially increased and then decreased [28]. Nevertheless, species extinction caused by environmental stress is a relatively long-term process in a given ecosystem [29]. In the short term, although changes in species richness are minimal, variations in species composition within communities formed by different species may have a greater impact on phytoplankton biomass than changes in species richness.

The mixed culture system of multiple algal species constructed in previous research [17] could be used to study the response of phytoplankton communities to environmental changes in the short terms. However, the conclusions obtained from microcosm experiments are usually inconsistent with the results of field investigation. Therefore, it is necessary to mutually verify the results of field investigations and microcosm experiments. This work aimed to answer the following questions through a mixed culture of various algal species combined with a field investigation: (1) What are the responses of diversity indices based on ranking distribution as well as traditional diversity indices of the phytoplankton community to temperature and nutrient gradients when the number of species remains unchanged in the short term? (2) What is the correlation between co-occurrence network topological characteristics and the above diversity indices? (3) What is the relationship between the above diversity indices and phytoplankton biomass? Addressing the aforementioned questions will help us understand how phytoplankton diversity responds to environmental changes, and explore correlations between diversity and interspecific relationships within phytoplankton communities.

## Materials and methods

### Field investigations

#### Sampling areas

**Figure 1:**
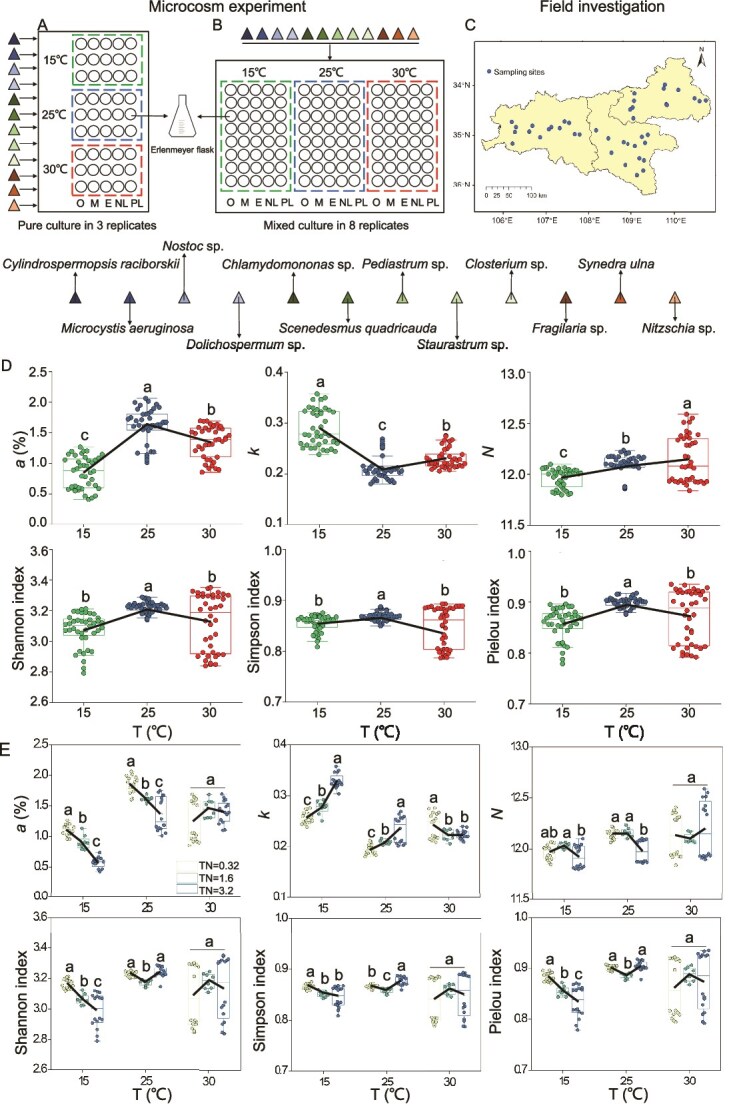
(A) Monoculture. (B) Mixed culture of microcosm experimental design. (C) Map of sampling points for field investigations. (D) Changes in the algal diversity index at different temperatures in microcosm experiments. (E) Changes in the algal diversity index in response to increasing concentrations of TN under different temperature conditions in microcosm experiments. Lines connecting the box plots indicate mean values. Tukey’s HSD test was used for multiple comparisons, and different lowercase letters indicate significant differences among treatments (*P* < .05).

The study area was the southern Qinling Mountains, covering Hanzhong City, Ankang City, and Shangluo City. This area (31°42′ ~34°24’N, 105°30′ ~111°1′E) is located in the south of Shaanxi Province, with a total area of 27 246 km^2^ (Fig. 1). Forty-one sampling sites were selected from 41 separate ponds. Sampling was carried out in four seasons from August 2019 to April 2021. Both phytoplankton and water samples were collected (n = 164 each).

#### Collection and determination of water samples

A plexiglass water sampler was used to collect 2 L of water samples 0.5 m below the water surface, which were packed into two 1-L polyethylene plastic bottles. One bottle of water sample was used to measure chlorophyll a (Chl a) and water quality, and the other one was fixed with 15mL of Lugol’s reagent for phytoplankton counting.

The total nitrogen (TN), total phosphorus (TP), and Chl a of the raw water samples were analyzed. TN and TP were measured according to the Standard Methods for the Examination of Water and Wastewater [30]. Chl a was determined according to the method described in previous research [31].

Phytoplankton samples were counted using the random field counting method. The phytoplankton sample was allowed to settle for 48 h. Afterwards, the supernatant was removed with a siphon tube, and the remaining 20–25 mL suspension was transferred to a 30mL quantification bottle. A little supernatant was used to wash the sediment residues three times and then was collected into the quantification bottle mentioned above. Finally, 0.5 mL of Lugol’s reagent was added and the sample volume was adjusted to 30mL with supernatant [32]. After thorough mixing, 0.1 mL of the fixed phytoplankton sample was immediately placed into a 20 mm × 20 mm phytoplankton counting chamber and covered with a coverslip, the sample was then counted under 10 × 20 magnification or 10 × 40 magnification using an optical microscope (CX31, Olympus, Tokyo, Japan). When the phytoplankton cell density was high, 200 phytoplankton counting was considered sufficient amount; if the phytoplankton cell density was too low, then the whole piece should be counted. The mean value of three counts for each phytoplankton sample was used for phytoplankton counting and identification. Phytoplankton species were identified according to previous research [33, 34].

### Microcosm experiment

#### Selection of algal strains

In the current study, 12 algal species were selected, including four Cyanobacteria species (*Cylindrospermopsis raciborskii*; *Microcystis aeruginosa*; *Nostoc* sp.; *Dolichospermum* sp.), five Chlorophyta species (*Chlamydomonas* sp.; *Scenedesmus quadricauda*; *Pediastrum* sp.; *Staurastrum* sp.; *Closterium* sp.) and three Bacillariophyta species (*Fragilaria* sp.; *Synedra ulna*; *Nitzschia* sp.). All the selected species were dominant in field investigations. The above algal species were purchased from the Freshwater Algae Culture Collection at the Institute of Hydrobiology, Chinese Academy of Sciences (Wuhan, China). The 12 algal species were monocultured in the modified BG11 + CSI (V_BG11_: V_CSI_ = 1: 1) medium at 25°C with a light intensity of 40 μmol photons m^−2^ s^−1^ and a light-to-darkness ratio of 12 h:12 h for 3 months before the experiment.

### Experimental design

The culture experiments were divided into two groups. In the first group, twelve species of algae were cultured separately. In the second group, these twelve species of algae were mixed at equal initial biomass for the mixed culture. In each group, algae were batch-cultured under five different nutrient conditions (oligotrophic, mesotrophic, eutrophic, nitrogen limitation, and phosphorus limitation) [35, 36] at three temperatures (15, 25, and 30°C) (Table S1). The concentrations of nitrogen and phosphorus were set to simulate oligotrophic, mesotrophic, and eutrophic conditions, as well as nitrogen and phosphorus limitation, according to the Organization for Economic Co-operation and Development (OECD) [37]. The optimal N:P ratio was set as described by Reynolds [36]. The monoculture and mixed culture were set up with 3 and 8 replicates, respectively. A total of 660 treatments were prepared (Fig. 1).

The modified BG11 + CSI (V_BG11_: V_CSI_ = 1: 1) medium without nitrogen and phosphorus was prepared as the culture medium of oligotrophic treatment, because the soil extract in CSI medium made the nitrogen and phosphorus concentrations in the modified culture medium reach 0.32 mg L^−1^ TN and 0.02 mg L^−1^ TP, respectively. This modified culture medium was also used as a base medium for other treatments including mesotrophic, eutrophic, nitrogen limitation, and phosphorus limitation treatments. The nitrogen and phosphorus concentrations of the culture medium in these treatments were adjusted according to Table S1 using 3.2 g L^−1^ NaNO_3_ and 0.2 g L^−1^ K_2_HPO_4_ solution prepared with the base medium. Erlenmeyer flasks (250 mL) containing 100 mL culture medium were autoclaved at 121°C for 30 min before inoculation. The initial algal biomass for inoculation for each treatment was 2.4 mg L^−1^. The microcosm experiments were conducted for 7 days at a light intensity of 40 μmol photons m^−2^ s^−1^ with a light–dark ratio of 12 h:12 h. Due to the different water evaporation rates at different temperatures, distilled water (autoclaved) was added every other day, and the total amount of distilled water added was 4.5, 7.75, and 14.5 mL at 15, 25, and 30°C, respectively.

At the end of the experiment, 10 mL of algal solution was taken in a 15mL centrifuge tube, then 1.5% Lugol’s reagent was added, and the algal community was counted using an optical microscope. The algal biomass was calculated based on the previous study [17]. The specific growth rate of algae was calculated based on the biomass [38]. The inhibition rate of algae was calculated based on the specific growth rate in mono- and mixed cultures according to equation (1):


(1) \begin{equation*} \mathrm{Ir}\left(\%\right)=\left(\left({\mathrm{\mu}}_{\mathrm{c}}-{\mathrm{\mu}}_{\mathrm{t}}\right)/{\mathrm{\mu}}_{\mathrm{c}}\right)\times 100 \end{equation*}

where Ir is the inhibition rate, μ_c_ and μ_t_ are the mean values of the mean growth rates of mono- and mixed cultures, respectively. The inhibition rate (Ir) quantifies the difference in a taxon’s growth rate between monoculture and mixed culture. A positive Ir indicates that the growth rate in the monoculture is higher than that in the mixed culture. Conversely, a negative Ir value implies that the growth of a taxon is promoted by the presence of other taxa in the mixed culture.

The treatments were categorized based on the mean biomass across all treatments. A treatment group was defined as being under non-stress conditions for algal growth if the ratio of its biomass to the mean biomass was ˃0.8. Conversely, it was defined as being under stress conditions if the ratio was ˂0.8. Accordingly, non-stress conditions comprised mesotrophic, eutrophic, nitrogen limitation, and phosphorus limitation at moderate temperature, as well as the same set of treatments at high temperature. Stress conditions comprised all treatments at low temperature, along with oligotrophic treatments at both moderate and high temperatures.

### Construction and characterization of co-occurrence networks

Community co-occurrence networks of the phytoplankton community data in the eight mixed cultures for all 15 treatments were analyzed. The “SparCC” package in R was used to calculate the pairwise correlations between the relative biomass of 12 algal species (Pearson correlation). The network visualization was plotted with Gephi version 0.10.1. The node represented the relative biomass of algae and the edge represented the correlation between two algal species.

The complexity and stability of co-occurrence networks were characterized using co-occurrence matrix-based interactive networks in order to investigate the stability of phytoplankton communities. Network complexity included closeness centrality, eigenvector centrality, weighted degree, positive connectedness, and |negative connectedness|. Closeness centrality is the reciprocal of the sum of the shortest path distances from a node to all other nodes, reflecting how closely a node is connected to others in the network. In other words, a high closeness centrality of a node implies that the average distance from a node to all other nodes is short. Nodes with high closeness centrality can typically propagate information and influence other nodes more efficiently. Closeness centrality can serve as a potential indicator for identifying keystone species, as nodes with high values are positioned to exert rapid and broad influence across the network. Eigenvector centrality is a network analysis metric that measures the importance of a node based on both the quantity and quality of its connections. It considers that a node’s influence depends on its neighbors’ importance and thus a node connected to highly central nodes has a high value of eigenvector centrality [39]. Weighted degree represents the sum of weights of all edges connected to a node, providing a more nuanced measure than simple degree counts [40]. Positive and negative connectivity are the sums of positive and negative edge weights, respectively, reflecting potential cooperative and competitive interactions within the network [41]. The complex topological characteristics of the network were considered to be the complexity of species interactions between algae [42]. Subsequently, the cohesion value of a network was calculated using positive and negative connectedness to characterize its stability [43]. Cohesion provides insights into the associations among taxa. Positive cohesion ranges from 0 to 1. Higher positive cohesion values reflect stronger positive statistical associations among taxa, which may arise from shared environmental responses or interactions. Negative cohesion ranges from −1 to 0. Higher absolute values of negative cohesion reflect stronger negative statistical associations among taxa, which may stem from balanced constraints formed by antagonistic processes such as competitive exclusion among species. Total cohesion is the sum of the absolute values of positive and negative cohesion, thereby reflecting the stability of a network [43]. For each community, the cohesion value was calculated as the sum of the connectedness of the community taxa, weighted by their respective biomass. As positive and negative connectedness were calculated separately, there were two cohesion values, one positive and one negative [21].

### Statistical analysis

The indices *a*, *k*, and *N* proposed by previous research were calculated based on the relative abundance or biomass of phytoplankton as follows [31]: (1) Calculate the percentage of each phytoplankton genus (*P_i_*) within a community from cell density or biomass data. (2) Rank these percentages from the smallest to the largest. (3) Draw a scatter plot of rank numbers (*i*) against percentages of phytoplankton genera and fit it with the exponential function (*P_i_* = *a* × *e^ki^*). (4) Obtain values of *a* and *k* from the fitted function and calculate *N* using equation (2).


(2) \begin{equation*} \frac{a{e}^k\left(1-{e}^{kN}\right)}{1-{e}^k}=100\% \end{equation*}

where *i* is the ranked number of each genus in a phytoplankton community, *P_i_* is the calculated percentage of each phytoplankton genus, *e* is the mathematical constant, both *a* and *k* are parameters derived from the exponential fit, and *N* is calculated using equation (2).

The calculated *N* indicates the theoretical maximum number of genera that can be supported by the environment where the phytoplankton samples were collected. In natural ecosystems, the observed genus richness may not exceed *N*. The index *a* is the percentage of genus abundance when the genus ranking number is 0. The index *a* represents the minimum percentage threshold required for a genus to persist within a community. If a genus’s percentage falls below *a*, it faces a high risk of local extinction. Thus, the index *a* indicates the critical percentage of taxon extinction. The index *k* is in fact the slope of the exponential function used to fit the data. When *k* is large, some genera are extremely dominant and a few genera have low abundance. In contrast, more genera have low abundance and a few genera are dominant. Thus, the index *k* indicates the evenness of the taxon distribution. The traditional diversity indices were calculated according to phytoplankton abundance or biomass separately, including Shannon [44], Simpson [45], and Pielou indices [46].

The trophic state index (TSI) is a quantitative indicator for assessing water eutrophication levels, integrating key parameters including chlorophyll a (Chl a), total nitrogen (TN), and total phosphorus (TP) to comprehensively evaluate aquatic trophic status. In this study, TSI values were calculated according to a previously published method [47], as detailed in equations (3)–(6).


(3) \begin{equation*} \mathrm{TSI}=0.421\mathrm{TSI}\left(\mathrm{Chl}\ \mathrm{a}\right)+0.282\mathrm{TSI}\left(\mathrm{TN}\right)+0.297\mathrm{TSI}\left(\mathrm{TP}\right) \end{equation*}

(4) \begin{equation*} \mathrm{TSI}\left(\mathrm{Chl}\ \mathrm{a}\right)=10\times \left(2.5+1.086\mathrm{lnChl}\ \mathrm{a}\right) \end{equation*}

(5) \begin{equation*} \mathrm{TSI}\left(\mathrm{TP}\right)=10\times \left(9.436+1.624\mathrm{lnTP}\right) \end{equation*}

(6) \begin{equation*} \mathrm{TSI}\left(\mathrm{TN}\right)=10\times \left(5.453+1.624\mathrm{lnTN}\right) \end{equation*}

where TSI (Chl a), TSI (TN), and TSI (TP) are the trophic state indices based on Chl a, TN, and TP, respectively. The TSI classification thresholds were defined as follows: oligotrophic (TSI ≤ 30), mesotrophic (30 < TSI ≤ 50), lightly eutrophic (50 < TSI ≤ 60), and medium-high eutrophic (TSI > 60).

Redundancy analysis (RDA) was performed using the “vegan” package in R to assess the effects of environmental factors on phytoplankton biomass and network stability. Additionally, a biomass weighted cohesion index was constructed based on biomass and negative cohesion to effectively differentiate among the treatment conditions according to Equation (7).


(7) \begin{equation*} \mathrm{BWC}=\frac{\mathrm{B}}{{\mathrm{B}}_{\mathrm{mean}}}\times \mid \mathrm{NC}\mid \end{equation*}

where BWC is the biomass weighted cohesion, B is the phytoplankton community biomass under a specific treatment, B_mean_ is the mean biomass of the eight parallel samples for that treatment, and |NC| is the absolute value of negative cohesion (|negative cohesion|) of the phytoplankton for the treatment.

The relative contributions of temperature (T), TN, and TP to phytoplankton biomass, diversity indices, and co-occurrence network stability were determined by variance partitioning analysis (VPA) using the “MuMIn” package. Partial least squares structural equation modeling (PLS-SEM) was applied to quantify the direct and indirect effects of nutrients and cohesion on phytoplankton diversity indices (*a*, *k*, and *N*), using the “plspm” package for the modeling, and the goodness-of-fit index (GOF) was used to assess the overall fit of the model.

Sampling maps were generated using ArcGIS (v.11.2). Multiple comparisons were performed using Tukey’s honestly significant difference (HSD) test.The relationships between diversity indices and both network cohesion and biomass, as well as between network topological characteristics and specific growth rate inhibition across temperature and nutrient gradients, were analyzed using linear fitting via the ordinary least squares (OLS) method. Data were processed in Microsoft Excel and visualized using Origin 2023.

**Table 1:** The relationship between diversity indices and environmental gradient (T, TN, and TP) under microcosm experiments and field investigations. (The mean temperatures in autumn and summer were 15.6°C and 28.4°C, respectively).

	Microcosm experiments				Field investigations			
		*a*	*k*	*N*		*a*	*k*	*N*
T (°C)	TN = 0.32	∩	∪	↑↑↑	TN < 0.96	∩	↓	∪
	TN = 1.6	∩	∪	∩	0.96 ≤ TN < 2.4	↑	↓	∪
	TN = 3.2	↑↑↑	↓↓↓	↑↑↑	TN ≥ 2.4	↑	↓	—
	TP = 0.02	∩	↓↓↓	↑↑↑	TP < 0.06	∩	↓	∪
	TP = 0.1	∩	∪	∩	0.96 ≤ TP < 0.15	↑	↓	∪
	TP = 0.2	↑↑↑	↓↓↓	—	TP ≥ 0.15	↑	↓	—
TN (mg L^−1^)	T = 15°C	↓↓↓	↑↑↑	∩	T = 15.6°C	↓↓↓	↑	↓
	T = 30°C	—	↓↓↓	—	T = 28.4°C	—	—	—
TP (mg L^−1^)	T = 15°C	—	—	↑	T = 15.6°C	—	—	—
	T = 30°C	—	—	—	T = 28.4°C	↓	—	↑

Note: ∩: increase then decrease; ∪: decrease then increase; ↑↑↑: significant increase; ↓↓↓: significant decrease; ↑: increase but not significant; ↓: decrease but not significant; —: no clear trend. Highlighting indicates consistent results between microcosm experiments and field investigations.

## Results

### Effects of temperatures and nutrient gradients on algal diversity index

Three diversity indices (*a*, *k*, and *N*) based on ranking distribution and three traditional diversity indices (Shannon, Simpson, and Pielou indices) were employed to evaluate the response of phytoplankton community and diversity to temperature and nutrient gradients. The critical percentage of taxon extinction *a* represents the minimum percentage threshold required for a taxon (e.g. a genus) to survive within the community. A decreasing value of *a* indicates an increasing risk of local extinction for certain taxa. The index *k* is the slope of the exponential function used to fit the data, reflecting the evenness of taxon distribution. An increasing *k* value implies a decreasing evenness and indicates that the community is dominated by a few highly abundant taxa. The index *N* indicates the theoretical maximum number of taxa that can be supported by the ecosystem where the phytoplankton samples were collected and is referred to as the theoretical maximum number of taxa.

The values of the *a*, Shannon, Simpson, and Pielou indices were the largest, whereas *k* was the lowest at a moderate temperature (Figs 1 and S1), suggesting that a moderate temperature was more conducive to phytoplankton achieving higher taxon maintenance capacity, evenness, and diversity. No significant differences were found in any diversity indices across treatments with varying nitrogen or phosphorus concentrations (Figs S2 and S3). However, the index *a* decreased but *k* increased with increasing nitrogen concentrations at both 15°C and 25°C (Fig. S2), implying that increasing nitrogen concentrations may raise the extinction risk for some taxa and reduce community evenness at low and moderate temperatures. In addition, the values of all three traditional diversity indices were the highest at the moderate N:P ratio (16:1) (Fig. S4).

**Figure 2:**
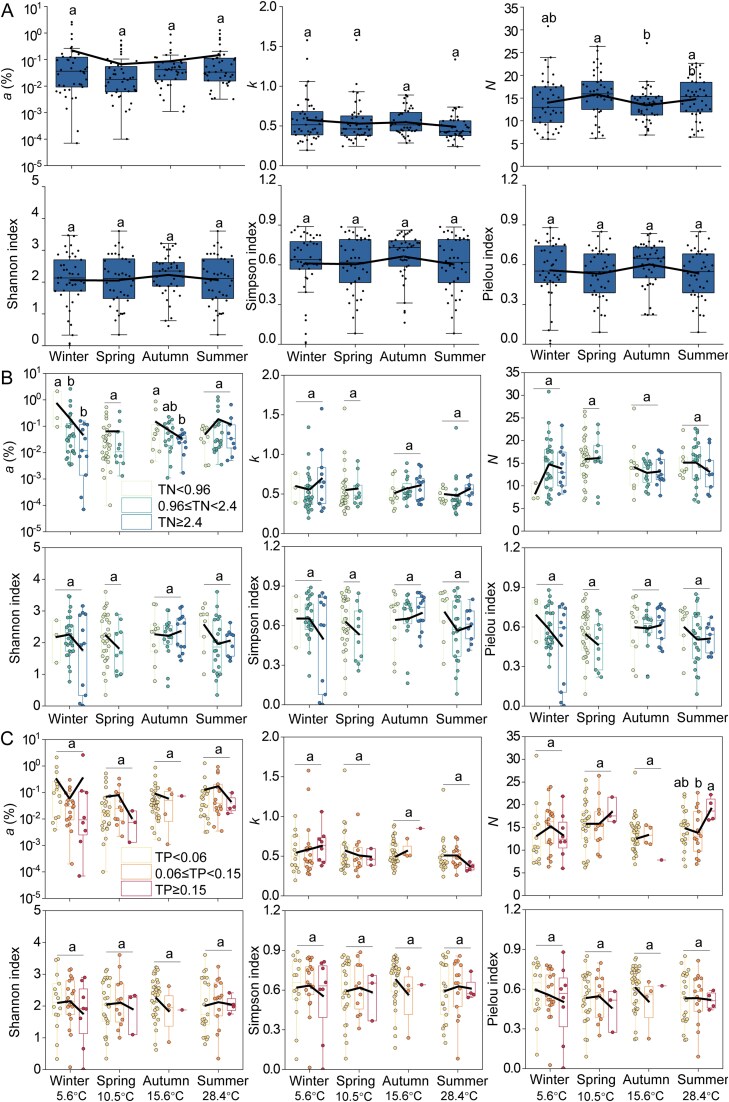
Changes in the algal diversity index in response to (A) temperature (T), (B) total nitrogen (TN), and (C) total phosphorus (TP) in field investigations. The lines between the box plots are the lines of the mean values. Tukey’s HSD test was used for multiple comparisons, and different lowercase letters indicate significant differences among treatments (*P* < 0.05).

An integrated analysis of microcosm experiments and field investigations revealed broadly consistent patterns in the responses of phytoplankton diversity indices (*a*, *k*, and *N*) to temperature and nutrient gradients (Table 1). Under low-nutrient conditions (microcosm: TN ≤ 1.6mg·L^−1^, TP ≤ 0.1 mg·L^−1^; field: TN < 0.96 mg·L^−1^, TP < 0.06 mg·L^−1^), index *a* exhibited a unimodal response to temperature in both experimental and field datasets, with the highest values occurring at intermediate temperatures (Figs S2, S3, S9, and  S10). Under high-nutrient conditions (microcosm: TN = 3.2 mg·L^−1^, TP = 0.2 mg·L^−1^; field: TN ≥ 2.4 mg·L^−1^, TP ≥ 0.15 mg·L^−1^), rising temperature consistently led to an increase in *a* and a decrease in *k* in both systems, indicating similar shifts in taxon maintenance capacity and community evenness across controlled and natural environments (Figs S2, S3, S9, and  S10). Furthermore, at low temperatures (microcosm: 15°C; field: mean spring temperature 15.6°C), index *a* decreased whereas index *k* increased with rising TN concentrations in both microcosm experiments and field investigations, suggesting comparable diversity responses to nitrogen enrichment under cold conditions (Figs 1 and 2). These results indicate a high degree of consistency in the direction of diversity responses to interacting temperature and nutrient gradients between microcosm experiments and field investigations. In contrast, the response of index *N* differed between microcosm experiments and field investigations. In microcosm experiments, the total number of taxa was fixed at 12, so *N* remained approximately constant. In field investigations, species richness varied substantially across sites and seasons, resulting in a wider range of *N* values compared to the microcosm experiments and producing a U-shaped relationship with temperature. Overall, at high nitrogen and phosphorus concentrations, index *a* increased whereas index *k* decreased with rising temperature; conversely, under low-temperature conditions, increasing total nitrogen concentrations led to a decrease in *a* and an increase in *k*, highlighting the significant interactive effects of temperature and nutrients in shaping phytoplankton diversity.

### Relationship between diversity index and standing stock

**Figure 3:**
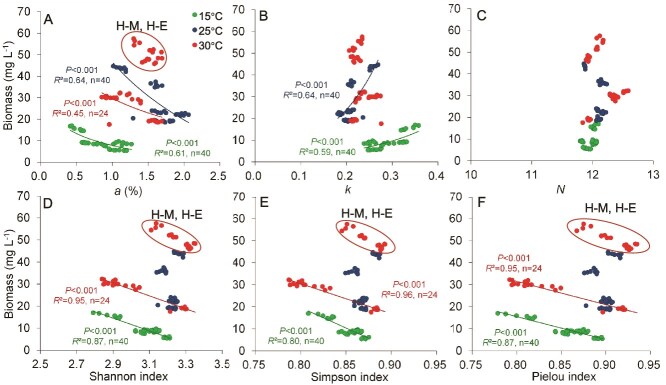
Changes in biomass of algal communities in relation to diversity indices. Relationships between phytoplankton biomass and (A) *a*, (B) *k*, (C) *N*, (D) Shannon index, (E) Simpson index, and (F) Pielou index under three temperature treatments. H-M and H-E were mesotrophic and eutrophic treatments at high temperatures, respectively.

To clarify the relationship between diversity indices and standing stock (biomass), the current study systematically analyzed the response relationship between the diversity indices (including *a*, *k*, *N*, Shannon, Simpson, and Pielou indices) and standing stock (biomass) under different temperature gradients through microcosm experiments. There was a significant linear relationship between diversity indices and biomass at different temperatures (Fig. 3). Specifically, index *a* showed a significant negative correlation with biomass, except under high-temperature mesotrophic and eutrophic conditions. There was a significant negative correlation between the traditional diversity indices and biomass (*R*^2^ > 0.8, *P* < 0.001) under all low-temperature nutrient gradients, as well as under high-temperature oligotrophic, nitrogen limitation, and phosphorus limitation conditions. In contrast, there was no significant linear relationship between the traditional diversity indices and biomass at moderate temperatures.

### Relationship between the stability of co-occurrence networks and diversity index

In the current study, co-occurrence network cohesion (including positive, negative, and total cohesion) is used as a structural proxy to infer the potential stability of phytoplankton communities. Network cohesion reflects the intensity and balance of species interactions, serving as an important feature of community structure [53]. Higher cohesion may enhance network stability, improving its ability to adapt to and resist environmental fluctuations and species competition [43].

To compare the co-occurrence patterns of 12 algal species with temperatures (15, 25, and 30°C) and nutrient gradients (oligotrophic, mesotrophic, eutrophic, nitrogen limitation, and phosphorus limitation), 15 co-occurrence networks (Fig. S11) were constructed. The node size of the co-occurrence network represented the relative biomass in each community. At low temperature (15°C), the relative biomass of *Fragilaria* sp. was the highest, and the number of positive correlation edges in the network exceeded that of negative correlation edges across all nutrient gradients except for the phosphorus limitation condition. At moderate temperature (25°C), the dominant species shifted to *Staurastrum* sp., with positive correlation edges similarly dominating, except under mesotrophic and phosphorus limitation conditions. At high temperature (30°C), *Nostoc* sp. and *Staurastrum* sp. were co-dominant species, and the number of positive correlation edges was significantly greater than the number of negative correlation edges in all five nutrient gradients, indicating that positive interactions were generally enhanced under high-temperature conditions. Moreover, co-occurrence networks of phytoplankton were constructed in ponds of the southern Qinling Mountains across seasons and trophic state gradients (Fig. S12). Networks in all four seasons exhibited high complexity, with positive correlations dominating in spring and winter, whereas stronger competitive interactions were observed in summer and autumn. Along the TSI gradient, the number of network connections increased with rising TSI. In particular, when TSI ≥ 60, node sizes enlarged and several key taxa occupied central positions, indicating that higher trophic status enhanced the connectivity and interaction intensity of phytoplankton communities.

**Figure 4:**
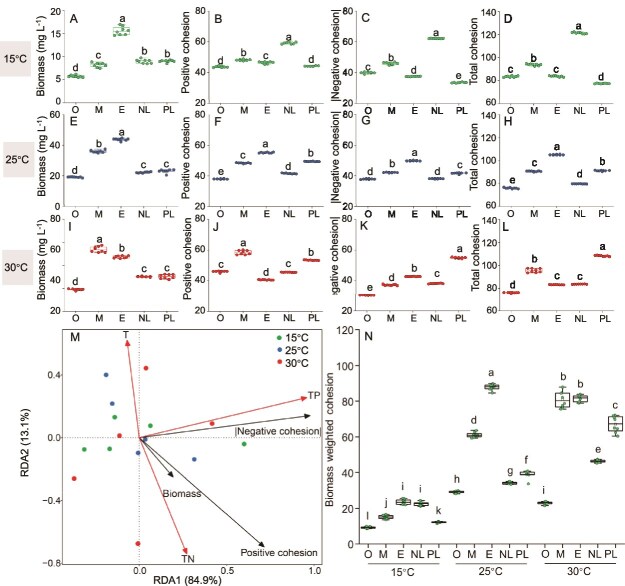
Differences in biomass, positive cohesion, |negative cohesion|, and total cohesion across different nutrient gradients under (A, B, C, and D) low, (E, F, G, and H) moderate, and (I, J, K, and L) high temperature conditions in microcosm experiments. (M) Redundancy analysis (RDA) of algae cohesion and biomass in relation to environmental factors (T, TN, and TP). (N) Variations in the biomass weighted cohesion across different temperature and nutrient gradients. Lowercase letters indicate statistically significant differences (*P* < 0.05).

Biomass and cohesion (including positive, negative, and total cohesion) varied under different temperature and nutrient gradients (Fig. 4). The current study conducted an RDA of biomass and cohesion in relation to T, TN, and TP to effectively distinguish all 15 treatment conditions. The results revealed a weak correlation between biomass and |negative cohesion|. Consequently, a biomass weighted cohesion index was established using these two indicators. This index effectively differentiated the treatment conditions, successfully classifying the 15 treatments into 12 significantly distinct gradients (Fig. 4).

**Figure 5:**
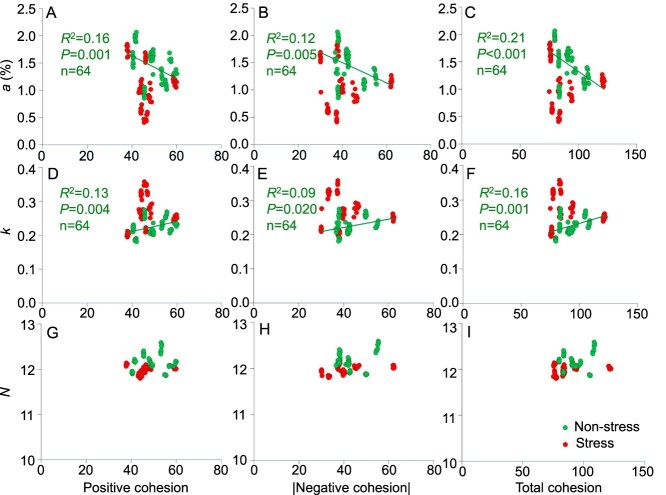
Relationships between co-occurrence network cohesion values and diversity indices (*a*, *k*, and *N*). The 15 treatments were divided into two groups: One represented dots under non-stress conditions for algal growth (including four nutrient gradients—Mesotrophic, eutrophic, nitrogen limitation, and phosphorus limitation—at both moderate and high temperatures), and the other represented dots under stress conditions for algal growth (including all low-temperature nutrient treatments, as well as oligotrophic conditions at both moderate and high temperatures). *N* was not fitted in this analysis as no species extinctions were observed during the short-term experiment and its variation was limited under the experimental conditions.

To investigate the relationship between co-occurrence network topological characteristics and the diversity of phytoplankton communities, this study systematically analyzed relationships between topological characteristics (positive cohesion, negative cohesion, and total cohesion) and diversity indices (*a*, *k*, *N*, Shannon, Simpson, and Pielou indices) under different treatment conditions (Figs 5 and S15, Table S2). A total of 15 treatments were divided into two groups at different temperatures (15, 25, and 30°C) and nutrient gradients (oligotrophic, mesotrophic, eutrophic, nitrogen limitation, and phosphorus limitation). The first group was treated as non-stress conditions for algal growth (including four nutrient gradients—mesotrophic, eutrophic, nitrogen limitation, and phosphorus limitation—at both moderate and high temperatures). The other group was treated as stress conditions for algal growth (including all low-temperature nutrient treatments, as well as oligotrophic conditions at both moderate and high temperatures).

Under non-stress conditions for algal growth, *a* showed a significant negative correlation with cohesion [positive cohesion (*R*^2^ = 0.16, *P* = 0.001), |negative cohesion| (*R*^2^ = 0.12, *P* = 0.005) and total cohesion (*R*^2^ = 0.21, *P* < .001)]. Conversely, *k* showed a significant positive correlation with cohesion [positive cohesion (*R*^2^ = 0.13, *P* = 0.004), |negative cohesion| (*R*^2^ = 0.09, *P* = 0.02), and total cohesion (*R*^2^ = 0.16, *P* = 0.001)] (Fig. 5). Under stress conditions, there was no significant linear relationship between the traditional diversity indices and the cohesion values. Furthermore, these indices did not correlate well with the connectivity and stability of phytoplankton co-occurrence networks (Fig. S15). There was no significant correlation between the traditional diversity indices (Shannon, Simpson, and Pielou indices) and network cohesion (positive cohesion, |negative cohesion|, and total cohesion) (Fig. S16). In the field investigation, the index *a* was positively correlated with positive cohesion and total cohesion in winter (Fig. S16A). The index *k* showed a positive correlation with positive cohesion and total cohesion, whereas index *N* was negatively correlated with |negative cohesion| and total cohesion in spring. Regarding traditional diversity indices (Shannon, Simpson, and Pielou indices), they showed a significant positive correlation with positive cohesion in winter, but exhibited significant negative correlations with both positive and total cohesion in autumn.

### Relationship between co-occurrence network topological characteristics and algal inhibition rate

Based on the relationship of co-occurrence networks under temperature and nutrient gradients in microcosm experiments, the topological characteristics of the co-occurrence network were calculated as closeness centrality, eigenvector centrality, weighted degree, positive connectedness, and |negative connectedness| (Fig. S13). Under low-temperature conditions, the value of closeness centrality was significantly higher under oligotrophic conditions than under mesotrophic and eutrophic conditions (Fig. S13A). The weighted degree was higher under nutrient limitation conditions with either low or high temperatures (Fig. S13G and I), and it was also higher under mesotrophic and eutrophic conditions at moderate temperatures (Fig. S13H). With temperature and nutrient gradients changing, positive connectedness and |negative connectedness| showed a similar changing pattern (Fig. S13J–O). Moreover, the topological characteristics of phytoplankton co-occurrence networks in ponds of the southern Qinling Mountains varied significantly across seasons (Fig. S14A). Higher closeness centrality in summer and autumn indicated tighter associations and stronger interactions among species, whereas lower values in spring and winter reflected relatively loose community structures and weaker interspecific interactions. Significant differences in phytoplankton co-occurrence network topological characteristics were also observed in different TSI in summer (Fig. S14B). Under TSI < 30, closeness centrality, positive connectedness, and |negative connectedness| were all significantly higher than those under TSI ≥ 30. These results suggest that under nutrient-limited conditions, phytoplankton communities may strengthen interspecific cooperation or promote niche differentiation, thereby forming more compact and well-defined interaction networks.

Specific growth rates of each algal species differed between pure culture (PC) and mixed culture (MC) conditions, and these responses varied across temperature and nutrient gradients (Fig. S17). Under low temperature conditions, the specific growth rate of *M. aeruginosa* remained low in both pure and mixed cultures. Furthermore, in the mixed culture, the growth of *Dolichospermum* sp., *Closterium* sp., and *Nitzschia* sp. was inhibited, exhibiting negative specific growth rates. In contrast, the specific growth rates in mixed cultures were significantly higher than those in their respective pure cultures for the low temperature group (*M. aeruginosa*, *Staurastrum* sp., and *Fragilaria* sp.), the moderate temperature group (*Nostoc* sp. and *Staurastrum* sp.), and the high temperature group (*C. raciborskii*, *M. aeruginosa*, *Nostoc* sp., *S. quadricauda*, *Staurastrum* sp., and *Fragilaria* sp.).

**Figure 6:**
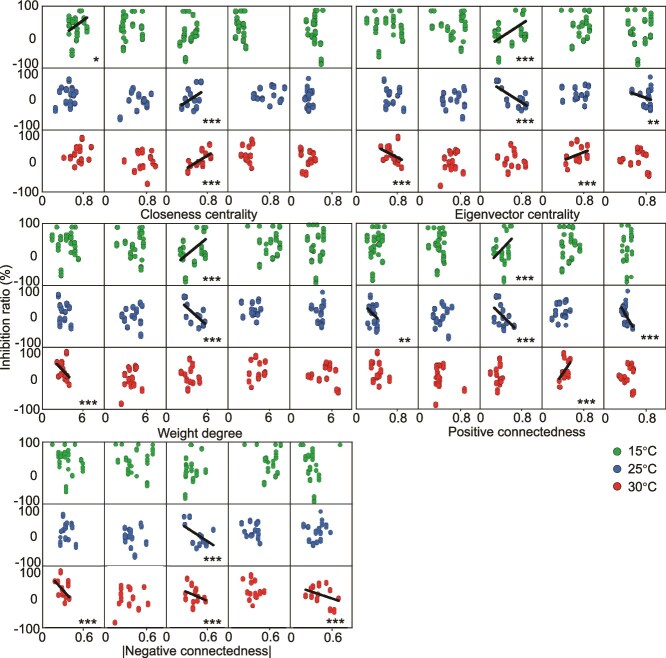
Relationships between network topological characteristics and inhibition rate of the specific growth rate under different temperature and nutrient gradients. (^*^P < .05, ^**^P < .01, and ^***^P < .001).

According to the algal biomass in mono- and mixed cultures, the inhibition rate of each algal species was calculated to illustrate the relationship between the network topological characteristics and the inhibition rate under temperature and nutrient gradients (Figs 6 and S18). The eigenvector centrality showed a significantly positive correlation with the inhibition rate under eutrophic conditions at low temperature and nitrogen limitation conditions at high temperature but exhibited a negative correlation under eutrophic and phosphorus limitation conditions at moderate temperature as well as under oligotrophic conditions at high temperature. Positive connectedness was positively correlated with inhibition rate under eutrophic conditions at low temperatures and under nitrogen limitation at high temperatures, and it were negatively correlated under oligotrophic, eutrophic, and phosphorus limitation conditions at moderate temperatures. |Negative connectedness| was negatively correlated with inhibition rate under eutrophic conditions at moderate temperatures and under oligotrophic, eutrophic, and phosphorus limitation conditions at high temperatures.

### The regulation of environmental gradients and cohesion values on diversity indices

In microcosm experiments and field investigations, the explanatory power of T, TN, and TP for phytoplankton biomass, diversity indices, and cohesion values differed (Fig. S19). In microcosm experiments, temperature explained over 20% of the variance in biomass, *a*, *k*, and *N*. It showed the greatest explanatory power for biomass (59%), followed by TN. In field experiments, temperature showed relatively high explanatory power for both positive cohesion (32%) and negative cohesion (12%). These results indicate that temperature was a key regulator of phytoplankton community characteristics in both microcosm experiments and natural environments.

**Figure 7:**
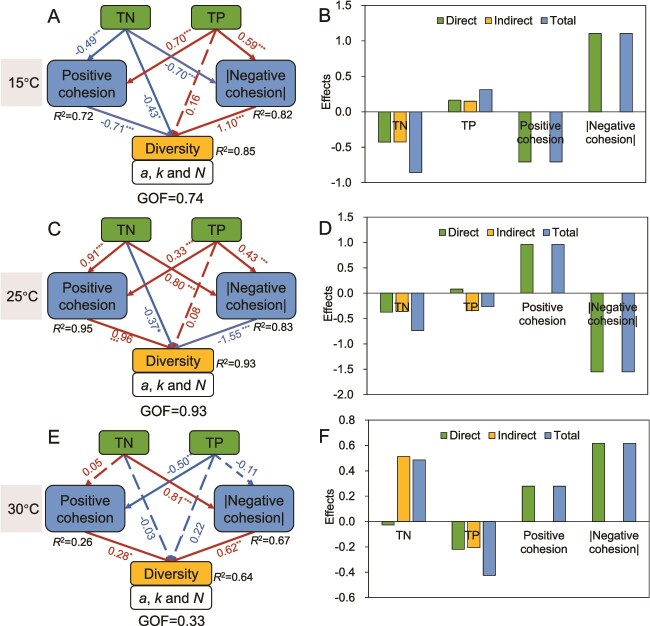
The partial least squares path model (PLS-PM) delineates the direct and indirect effects of nutrient (TN and TP) and cohesion values (positive cohesion and |negative cohesion|) on diversity indices (*a*, *k*, and *N*) under (A, B) low, (C, D) moderate, and (E, F) high temperature conditions in microcosm experiments. Red and blue arrows indicate positive and negative effects, respectively, with values adjacent to the arrows corresponding to standardized path coefficients. Solid lines represent statistically significant paths, whereas dashed lines represent non-significant paths. GOF represents the goodness-of-fit index for the overall model (^*^P < .05, ^**^P < .01, and ^***^P < .001).

Structural equation models (SEMs) were developed to investigate how nutrients and network stability affect phytoplankton diversity, based on data from microcosm experiments (Fig. 7) and field investigations (Fig. S20) across different temperature and seasonal gradients. In microcosm experiments, the goodness-of-fit indices of the SEMs under low, moderate, and high temperature conditions were 0.74, 0.93, and 0.33, respectively. As temperature increased, the direct negative effect of TN on diversity indices gradually weakened, with path coefficients shifting from −0.43 (*P* < 0.05) at low temperature to −0.37 (*P* < 0.05) at moderate temperature, and further to −0.03 (*P* > 0.05) at high temperature. In contrast, the indirect effect of TN on diversity transitioned from negative to positive. Moreover, both positive and negative cohesion exhibited significant positive effects on diversity indices only under high temperature conditions. In field investigations, the SEM for spring demonstrated the highest goodness-of-fit (0.36), whereas models for other seasons showed relatively lower fit. During spring, TP had a significant negative effect on diversity indices (path coefficient = −0.32, *P* < 0.05), whereas both positive and negative cohesion showed significant positive effects on diversity.

## Discussion

### Response of diversity indices to environmental gradients

Microcosm experiments in the current study showed that the *a* value (the minimum percentage threshold required for a taxon to survive) of the ranking distribution initially increased and then decreased with increasing temperature, whereas the *k* value (the evenness of the taxon distribution) exhibited an opposite trend. Field investigations showed that the *a* value usually had the highest value in summer and autumn, whereas the *k* value was lowest in summer within the same TSI group (Fig. S5). As the nutrient level increased, especially the nitrogen concentration, the *a* value decreased whereas the *k* value increased in microcosm experiments. With increase of TSI, the *a* value decreased and the *k* value did not change obviously in field investigations (Fig. S5). Overall, the variations found in microcosm experiments were basically consistent with those found in field investigations. Therefore, it could be concluded that the *a* value initially increased and then decreased with increase of temperature but decreased with increase of nutrient levels. The change of the *k* value was opposite to the *a* value.

Our results showed that the minimum value of *k* appeared at moderate temperatures and under high nutrient conditions. This environmental condition was suitable for the growth of various algal species. Under such circumstances, the majority of algal species were able to proliferate quickly [48]. Due to the consumption of resources by various species, the growth of the dominant species was limited [49, 50]. When the exponential model was used to fit the ranking distribution of the phytoplankton community, the *k* value was the slope of logarithms at both ends of the fitting formula. The smaller the *k* value, the smaller the relative biomass proportion of the dominant species and the larger the *a* value (the lesser the possibility of extinction of rare species) [17]. This exactly reflected that the habitat was suitable for the optimal growth of various algal species when the value of *k* was at its minimum. The traditional diversity indices (Shannon, Simpson, and Pielou indices) in microcosm experiments of the current study reached the maximum values at moderate temperature. The response of these indices to nutrient gradients was not obvious because the temperature had a more significant effect on algal diversity in microcosm experiments. Field investigations also demonstrated similar results to those from microcosm experiments, showing significant differences in diversity indices among various seasons but no considerable change with nutrient variations (Figs S5, S6, S7 and S8). As species richness was constant in microcosm experiments, a higher traditional diversity index indicated a higher evenness of the algal community. Higher evenness also suggested that most of the algal species could grow optimally at moderate temperatures and under high nutrient conditions [51]. In summary, under relatively favorable conditions for algal proliferation, the dominant species were weakened and rare species had more opportunities to proliferate, ultimately resulting in higher *a* values and lower *k* values.

### Relationship between topological characteristics of co-occurrence networks and diversity indices

The cohesion value in co-occurrence networks quantifies the degree to which various species are connected in a community by assessing the connections, including positive and negative interactions, between species in a community [43, 52]. Total cohesion is the sum of the absolute values of positive and negative cohesion. An increase in the former indicates greater cooperation among species, which helps the community resist environmental disturbance [53]. An increase in the absolute value of the latter indicates greater niche differentiation of species, which helps to enhance the community’s ability to resist interference by increasing redundancy [53]. Therefore, an increase in the sum of the two, that is, in total cohesion, can represent an increase in network stability [43, 52, 54]. In the present study, the cohesion value had linear relationships with *a* and *k*, but the trend of the relationship depended on environmental factors.

Under non-stress conditions for algal growth (including four nutrient gradients—mesotrophic, eutrophic, nitrogen limitation, and phosphorus limitation—at both moderate and high temperatures), index *a* showed a significant negative correlation with the cohesion values (positive cohesion, |negative cohesion|, and total cohesion) (*P* < 0.05), whereas index *k* exhibited an opposite trend (Fig. 5). Additionally, in spring (e.g. suitable temperature, increased light), the *k* was positively correlated with cohesion (positive cohesion and total cohesion). This correlation pattern is the same as that observed under non-stress microcosm conditions. Under non-stress conditions, elevated levels of TN and TP provide a resource foundation for the rapid proliferation of dominant species, whereas suitable temperatures further enhance their metabolic and growth rates [55]. Dominant species with competitive advantages can utilize available resources more efficiently, rapidly proliferate and establish dominance. This process reduces the evenness of species distribution within the community, which is reflected in the model as an increase in the *k*. Furthermore, constrained by narrower niches and weaker competitive abilities, rare species are disadvantaged in resource competition, leading to a reduced proportion of biomass (decreased *a*). This process simultaneously enhances interspecific associations, including cooperative interactions and interspecific competition, manifested as increases in both positive cohesion and |negative cohesion|, collectively elevating the total cohesion of the community. It is worth noting that if this process continues (e.g. by extending the experimental duration), it may intensify competitive exclusion, leading to further polarization of the community structure. This shift in the relative importance of potential driving processes was statistically supported by SEM analyses across temperature and seasonal gradients (Fig. 7). Therefore, under non-stress conditions, the structure of phytoplankton communities shifts from being primarily associated with environmental stress to being dominated by interspecific interactions.

The topological characteristics of co-occurrence networks are usually used to indicate the positive and negative relationships among microbial species in communities [21]. For phytoplankton, this relationship is usually obtained by calculating inhibition rates based on mono- and mixed cultures [56]. We aimed to clarify these relationships by interpreting how specific network topological parameters reflect competitive, collaborative, and niche differentiation processes among phytoplankton species. However, significant correlations between several topological characteristics of co-occurrence networks and the inhibition rate were only obtained under eutrophic conditions in the current study.

The inhibition rate reflects the relative change in specific growth rate by quantifying the difference in growth rates of each species under monoculture and mixed-culture conditions, rather than direct inhibition effects. The five topological characteristic parameters were significantly correlated with the inhibition rate in the eutrophic treatment under moderate temperature. Among them, closeness centrality showed a significant positive correlation, indicating that species with higher closeness centrality are more likely to be influenced by other species in the community. This suggested that species with higher connectivity may be more responsive to interspecies interactions. Whereas the remaining four parameters were significantly negatively correlated. Under eutrophic conditions with low temperature, eigenvector centrality, weighted degree and positive connectedness were significantly positively correlated with inhibition rate. Under oligotrophic conditions with high temperature, eigenvector centrality, weighted degree and |negative connectedness| were negatively correlated with inhibition rate. There were also a few significant correlations in other treatments, but no discernible rules could be obtained. These results suggest that the relationship between topological characteristics and inhibition rate is context-dependent. The above results indicated that in a suitable environment, such as the eutrophic condition with moderate temperature in the current study, each index of topological characteristic can accurately reflect the relationship among species reflected by this index theoretically, such as competition, collaboration, and niche differentiation.

### Relationship between diversity index and phytoplankton standing stock

The phytoplankton biodiversity is considered to affect the biomass (standing stock) in aquatic ecosystems [27]. In the current study, the *k* value was significantly positively correlated with the algal biomass at low and moderate temperatures, whereas the *a* value was opposite. The higher *k* value, indicating lower community evenness, highlighted a greater competitive advantage for the dominant species, whereas the rare species were more likely to be endangered. The biomass productivity of the dominant species was much higher than that of the rare species, resulting in the highest productivity of the community. The Shannon, Simpson, and Pielou indices were negatively correlated with the algal biomass in all treatments except for the mesotrophic and eutrophic treatments with high temperatures. The above results indicate that the stronger the proliferation ability of the algal community under a specific richness, the lower the uniformity; and vice versa.

In previous research, 16 species of algae were selected for microcosm experiments involving mixed cultures with combinations of 1, 2, 4, 8, and 16 species, respectively, to study the relationship between the number of species and the productivity of the algal community [14]. They showed that chlorophyll a concentration increased with the increase of species richness at 15, 25, and 30°C. Our results did confirm that algal species richness was positively correlated with standing stock. Large-scale field investigations demonstrated that the relationship between productivity and diversity of phytoplankton had a unimodal pattern, that is, the productivity increased initially and then decreased with the increase of richness [28, 57, 58]. Grazing by zooplankton, as well as fish and others, could significantly reduce phytoplankton biomass and thus lower standing stock [28]. Moreover, in addition to phytoplankton richness, species composition (the relative proportion of each phytoplankton species in the community) affected the resource acquisition of various phytoplankton species. Competition among these phytoplankton for resources could also have an impact on standing stock. Our study aimed to gain insight into the latter question.

In the current study, the relationship between species composition and productivity of phytoplankton was studied under the condition of constant richness. The *k* value was significantly positively correlated with algal biomass, and the *a* value was opposite. Traditionally, in order to obtain a phytoplankton community close to the natural phytoplankton community, the phytoplankton samples are taken from the field and cultivated in the laboratory. Afterwards, the culture was used to study the response of mixed phytoplankton communities to the environmental gradients. As community composition varied widely among various studies, the traditional diversity indices were finally employed to evaluate the variation in the community. However, there was no single correspondence between the traditional diversity index and community composition. The same diversity index might be calculated from several different communities. Nevertheless, the phytoplankton species ranking distribution was used in the current study. The two indices *a* and *k*, which were calculated under a given number of species *N*, were in one-to-one correspondence with the algal community. This largely preserves information about the phytoplankton composition of the community. If the three diversity indices (*a*, *k,* and *N*) were given, a ranking species composition can be deduced backwards, but the traditional diversity indices cannot achieve the same function.

This study provided statistical evidence for the potential processes involved in phytoplankton community assembly and algal interactions through combined microcosm experiments and field investigations, with a particular focus on the influence of temperature and nutrient availability. Several limitations should be addressed in future research to improve this mechanism and promote its application: Although this study demonstrated that the exponential curve can be used to fit the relative abundance ranking distribution of phytoplankton genera and calculate three corresponding diversity indices, this approach and the derived indices are also considered potentially applicable for assessing community composition and diversity in other organisms. Future studies are needed to verify the applicability of this method to other taxonomic groups. Second, this work only considered abiotic factors such as temperature and nutrient concentration, but did not account for the predation of phytoplankton by zooplankton and fish, as well as the influence of bacteria and fungi on the composition of phytoplankton communities. The influence of these biotic factors needs to be further elucidated in subsequent research.

## Conclusion

The responses of phytoplankton to temperature and nutrient gradients were determined by combining field investigations and microcosm experiments. Our study found that the *a* value initially increased and then decreased with the increase of temperature but decreased with the increase of nutrient levels. The results could be attributed to the fact that the dominant degree of the dominant species will be weakened and the rare species will have more opportunities for proliferation under the conditions suitable for the growth of various algae. There was a linear relationship among cohesion values, *a* and *k*, but the trend of the relationship depends on environmental factors, with a decreasing *a* and an increasing k as cohesion increased under non-stress conditions for algal growth. The *k* value was significantly positively correlated with algal biomass, and the *a* value was opposite under specific species richness. There was no single correspondence between the traditional diversity index and community composition, and the same diversity index might be calculated from several different communities. The two indices *a* and *k*, which are calculated under a given number of species *N*, are in one-to-one correspondence with the algal community. This largely preserves information about the phytoplankton composition of the community. The present study further analyzed the diversity indices with standing stock and stability of the co-occurrence network, which provided new insights into phytoplankton community succession and ecological processes in aquatic ecosystems induced by climate change and anthropogenic activities.

## Supplementary Material

Supplementary_material-ISME5_wrag049

## Data accessibility

All data needed to evaluate the conclusions in the paper are present in the paper and/or the Supplementary. Data are available from the Figshare Repository: 10.6084/m9.figshare.31293997.
