# Supplementary material for: Ranking distribution reveals opposite shifts in evenness and survival thresholds of phytoplankton under environmental stress
Source: ISME J. 2026 Mar 10;20(1):wrag049. doi: 10.1093/ismejo/wrag049 (PMC13064661; doi:10.1093/ismejo/wrag049)
Supplement: Supplementary_material-ISME5_wrag049 [file supplementary_material-isme5_wrag049.docx]

The supplementary material

**Ranking distribution reveals opposite shifts in evenness and survival thresholds of phytoplankton under environmental stress**

Sisi Ye^a^^,d^, Li Gao^b^, Chao Chang^a^, Xinyi Zhang^a^, Yun Zhou^a^, Man Xiao^c^, Fang Yang^a^, Ming Li^a,*^

^a^State Key Laboratory of Soil and Water Conservation and Desertification Control, College of Natural Resources and Environment, Northwest A&F University, Yangling, Shaanxi, 712100, China

^b^Institute for Sustainable Industries and Liveable Cities, Victoria University, PO Box 14428, Melbourne, Victoria, 8001, Australia

^c^State Key Laboratory of Lake Science and Environment, Nanjing Institute of Geography and Limnology, Chinese Academy of Sciences, Nanjing, 210008, China

^d^College of Soil and Water Conservation Science and Engineering (Institute of Soil and Water Conservation), Northwest A&F University, Yangling, Shaanxi, 712100, China

**^*^Corresponding author**

Ming Li, lileaf@163.com; lileaf@nwsuaf.edu.cn

**Results**

**Effects of temperatures and nutrient gradients on algal diversity index**

To assess the effect of TSI on phytoplankton diversity indices in southern Qinling Mountain ponds in different seasons, we compared the values in each season under different TSI ranges (Fig. S5 and S6). The index *a* calculated based on relative biomass (*a*, mean value=0.12%) was lower than that calculated based on relative abundance (*a*, mean value=0.44%), whereas *k* was opposite (The mean value of *k* was 0.54 when calculated based on relative biomass and 0.34 when calculated based on relative abundance). The *N* calculated based on relative biomass and relative abundance were analogous. The index *a* calculated based on relative biomass decreased with increasing TSI, suggesting that eutrophication may increase the extinction risk for taxa. The calculated results based on relative abundance revealed that the index *a* in autumn was significantly higher than those in the other seasons when TSI exceeded 30, implying that phytoplankton taxa were more prone to extinction in autumn. The index *k* had no significant change with the increase of TSI. The index *N* calculated based on both relative biomass and abundance increased obviously with increasing TSI (Fig. S5). In addition, both were significantly lower in autumn than in the other seasons when TSI ranged from 50 to 60, indicating that the theoretical maximum number of taxa supported by the ecosystem was lower in autumn under eutrophic conditions. Shannon, Simpson, and Pielou indices did not change significantly with the increase of TSI (Fig. S6). The correlations of algal biomass (Fig. S7) and abundance (Fig. S8) in different seasons and TSI with the diversity index were further analyzed. The index *a* showed a negative correlation with TSI, whereas *N* showed a positive correlation based on biomass-calculated. There was a significant positive correlation between TSI and the traditional diversity index in summer, and a positive correlation between TSI and *k* in autumn. There was a significant negative correlation between TSI and diversity index in other seasons (Fig. S7A). The index *a* showed a negative correlation with TSI based on abundance-calculated. There was no significant correlation between the diversity index and temperature in different TSI based on abundance-calculated (Fig. S8). These results indicated that TSI and diversity indices were more significantly correlated during the same season.

**Table S1** Nitrogen and phosphorus concentrations under nutrient gradients

|  | TN (mg L^-1^) | TP (mg L^-1^) | N:P ratio |
| --- | --- | --- | --- |
| Oligotrophic | 0.32 | 0.02 | 16 |
| Mesotrophic | 1.6 | 0.1 | 16 |
| Eutrophic | 3.2 | 0.2 | 16 |
| Nitrogen limitation | 0.32 | 0.2 | 1.6 |
| Phosphorus limitation | 3.2 | 0.02 | 160 |

**Table S2** The relationship between diversity indices and network stability under microcosm experiments and field investigations.

|  |  |  | *a* (%) | *k* | *N* |
| --- | --- | --- | --- | --- | --- |
| Microcosm experiment | Non-stress | Positive cohesion | ↓↓ | ↑↑ | -- |
|  |  | \|Negative cohesion\| | ↓↓ | ↑ | -- |
|  |  | Total cohesion | ↓↓↓ | ↑↑ | -- |
|  | Stress | Positive cohesion | -- | -- | -- |
|  |  | \|Negative cohesion\| | -- | -- | -- |
|  |  | Total cohesion | -- | -- | -- |
| Field investigations | Spring | Positive cohesion | -- | ↑↑ | -- |
|  |  | \|Negative cohesion\| | -- | -- | ↓ |
|  |  | Total cohesion | -- | ↑↑ | ↓↓↓ |
|  | Winter | Positive cohesion | ↑↑ | -- | -- |
|  |  | \|Negative cohesion\| | -- | -- | -- |
|  |  | Total cohesion | ↑ | -- | -- |

Note: ↑, ↓, and -- indicate positive, negative, and non-significant correlations, respectively. The number of symbols denotes the statistical significance level (↑/↓: *P* < 0.05, ↑↑/↓↓: *P* < 0.01, ↑↑↑/↓↓↓: *P* < 0.001).

**Fig. S1** Changes in the algal diversity index (**A** *a*, **B** *k*, **C** *N*, **D** Shannon index, **E** Simpson index, and **F** Pielou index) in response to increasing concentrations of TP under different temperature conditions in microcosm experiments. Lines connecting the box plots indicate mean values. Tukey’s HSD test was used for multiple comparisons, and different lowercase letters indicate significant differences among treatments (*P* < 0.05).

**Fig. S2** Changes in the algal diversity index with different total nitrogen (TN) concentrations in microcosm experiments. **A** The algal diversity index under different TN concentrations. **B** The algal diversity index under different temperature gradients at a constant TN concentration. Lines connecting the box plots indicate mean values. Tukey’s HSD test was used for multiple comparisons, and different lowercase letters indicate significant differences among treatments (*P* < 0.05).

**Fig. S3** Changes in the algal diversity index at different total phosphorus (TP) concentrations in microcosm experiments. **A** The algal diversity index under different TP concentrations. **B** The algal diversity index under different temperature gradients at a constant TP concentration. Lines connecting the box plots indicate mean values. Tukey’s HSD test was used for multiple comparisons, and different lowercase letters indicate significant differences among treatments (*P* < 0.05).

**Fig. S4** Changes in the algal diversity index at different N:P ratios in microcosm experiments. **A** The algal diversity index under different N:P ratios. **B** The algal diversity index under different temperature gradients at a constant N:P ratio. Lines connecting the box plots indicate mean values. Tukey’s HSD test was used for multiple comparisons, and different lowercase letters indicate significant differences among treatments (*P* < 0.05).

**Fig. S5** The relationships among trophic state index (TSI) of southern Qinling Mountain ponds, **A** *a*, **C** *k* and **E** *N* calculated based on algal biomass, and the relationships among TSI of southern Qinling Mountain ponds, **B** *a*, **D** *k* and **F** *N* calculated based on algal abundance. Tukey’s HSD test was used for multiple comparisons, and different lowercase letters indicate significant differences among treatments (**P* <0.05, ***P* <0.01 and ****P* <0.001).

**Fig. S6** The relationship among trophic state index (TSI) of southern Qinling Mountain ponds, and **A** Richness, **B** Shannon index, **C** Simpson index and **D** Pielou index calculated based on algal biomass. Tukey’s HSD test was used for multiple comparisons, and different lowercase letters indicate significant differences among treatments (**P* <0.05, ***P* <0.01 and ****P* <0.001).

**Fig. S7** **A** Pearson correlation between TSI and diversity indices (calculated by relative biomass) in different seasons, and **B** Pearson correlation between water temperature and diversity indices (calculated by relative biomass) in the southern Qinling Mountain ponds (**P* <0.05, ***P* <0.01 and ****P* <0.001).

**Fig. S8** **A** Pearson correlation between TSI and diversity indices (calculated by relative abudance) in different seasons, and **B** Pearson correlation between water temperature and diversity indices (calculated by relative abudance) in the southern Qinling Mountain ponds (**P* <0.05, ***P* <0.01 and ****P* <0.001).

**Fig. S9** Changes in the algal diversity index at different total nitrogen (TN) concentrations in field investigations. **A** The algal diversity index under different TN concentration gradients. **B** The algal diversity index across different seasons under the same TN concentration gradient. Lines connecting the box plots indicate mean values. Tukey’s HSD test was used for multiple comparisons, and different lowercase letters indicate significant differences among treatments (*P* < 0.05).

**Fig. S10** Changes in the algal diversity index at different total phosphorus (TP) concentrations in filed investigations. **A** The algal diversity index under different TP concentration gradients. **B** The algal diversity index across different seasons under the same TP concentration gradient. Lines connecting the box plots indicate mean values. Tukey’s HSD test was used for multiple comparisons, and different lowercase letters indicate significant differences among treatments (*P* < 0.05).

**Fig. S11** Co-occurrence network structures of 12 algal species at temperatures and nutrient gradients. **A**, **D**, **G**, **J** and **M** were co-occurrence network diagrams under oligotrophic, mesotrophic, eutrophic, nitrogen limitation and phosphorus limitation at low temperature, respectively; **B**, **E**, **H**, **K** and **N** were co-occurrence network diagrams under oligotrophic, mesotrophic, eutrophic, nitrogen limitation and phosphorus limitation at moderate temperature, respectively; **C**, **F**, **I**, **L** and **O** were co-occurrence network structures under oligotrophic, mesotrophic, eutrophic, nitrogen limitation and phosphorus limitation at high temperature, respectively. The size of the node indicated the relative biomass of the algae, the color of the node corresponded to the network module it belonged to, and the lines between the nodes indicated the correlation between the two connected algal species, with the red line indicating a positive correlation and the green line representing a negative correlation, and the width of the line between the nodes indicated the size of the correlation coefficients. (Cyl.: *Cylindrospermopsis raciborskii*; Mic.: *Microcystis aeruginosa*; Nos.: *Nostoc* sp.; Dol.: *Dolichospermum* sp.; Chl.: *Chlamydomonas* sp.; Sce.: *Scenedesmus* *quadricauda*; Ped.: *Pediastrum* sp.: Sta.: *Staurastrum* sp.; Clo.: *Closterium* sp.; Fra.: *Fragilaria* sp.: Syn.: *Synedra* *ulna*; Nit.: *Nitzschia* sp.).

**Fig. S12** Co-occurrence networks of phytoplankton in filed investigations in **A** spring, **B** summer, **C** autumn, and **D** winter. Co-occurrence networks of phytoplankton in summer under the conditions of **E** TSI<30, **F** 30≤TSI<50, **G** 50≤TSI<60, and **H** TSI≥60. The size of the node indicated the relative biomass of the algae, the color of the node corresponded to the network module it belonged to, and the lines between the nodes indicated the correlation between the two connected algal species, with the red line indicating a positive correlation and the green line representing a negative correlation, and the width of the line between the nodes indicated the size of the correlation coefficients.

**Fig. S13** Topological characteristics of co-occurrence networks at temperature and nutrient gradients. **A**, **D**, **G**, **J**, and **M** were the topological characteristics under oligotrophic, mesotrophic, eutrophic, nitrogen limitation, and phosphorus limitation conditions at low temperature, respectively; **B**, **E**, **H**, **K,** and **N** were the topological characteristics under oligotrophic, mesotrophic, eutrophic, nitrogen limitation and phosphorus limitation conditions at moderate temperature, respectively; **C**, **F**, **I**, **L,** and **O** were the topological characteristics under oligotrophic, mesotrophic, eutrophic, nitrogen limitation and phosphorus limitation conditions at high temperature, respectively. Tukey’s HSD test was used for multiple comparisons, and different lowercase letters indicate significant differences among treatments (*P* < 0.05).

**Fig. S14** Topological characteristics of co-occurrence networks in in filed investigations in different **A** seasons and **B** TSI. Lowercase letters indicate statistically significant differences (*P* < 0.05).

**Fig. S15** Co-occurrence network cohesion were related to Shannon, Simpson, and Pielou indices, respectively. The 15 treatments were divided into two groups: one represented by green dots under non-stress conditions for algal growth (including mesotrophic, eutrophic, nitrogen, and phosphorus limitations at moderate temperature and mesotrophic, eutrophic, nitrogen, and phosphorus limitations at high temperature) and the other represented by red dots under stress conditions for algal growth (including all low-temperature nutrient treatments, oligotrophy at moderate temperature and high temperature).

**Fig. S16 A** The relationship between network cohesion and *a*, *k*, and *N*, and **B** its relationship with the Shannon, Simpson, and Pielou indices across seasons in filed investigations (**P* < 0.05; ***P* < 0.01; ****P* < 0.001).

**Fig. S17** Specific growth rates of each algal species under pure culture (PC) and mixed culture (MC) conditions across different temperature and nutrient gradients. Lowercase letters indicate statistically significant differences (*P* < 0.05) in growth rates between PC and MC treatments for each species.

**Fig. S18** Relationships between network topological characteristics and inhibition rate of specific growth rate under different temperature. **A** The relationship between weighted degree and inhibition rate under moderate temperature. **B** The relationship between eigenvector centrality and inhibition rate under moderate temperature. **C** The relationship between |negative connectedness| and inhibition rate under high temperature (****P* <0.001).

**Fig. S19** The explanatory power of temperature (T), total nitrogen (TN) concentration, and total phosphorus (TP) concentration on phytoplankton biomass, diversity index (including *a*, *k*, *N*, Shannon index, Simpson index, and Pielou index), and co-occurrence network stability (including positive cohesion, |negative cohesion|, and total cohesion) **A** in microcosm experiments and **B** in filed investigations.

**Fig. S20** The partial least squares path model (PLS-PM) delineates the direct and indirect effects of nutrient factors (TN and TP) and cohesion values (positive cohesion and |negative cohesion|) on diversity indices (*a*, *k* and *N*) in spring (**A**, **B**), summer (**C**, **D**), autumn (**E**, **F**) and winter (**G**, **H**) in filed investigations. Red and blue arrows indicate positive and negative effects, respectively, with values adjacent to the arrows corresponding to standardized path coefficients. Solid lines represent statistically significant paths, whereas dashed lines represent non-significant paths. GOF represents the goodness-of-fit index for the overall model (**P* <0.05, ***P* <0.01 and ****P* <0.001).
